# Supplementary material for: High Prolactin Concentration Induces Ovarian Granulosa Cell Oxidative Stress, Leading to Apoptosis Mediated by L-PRLR and S-PRLR
Source: Int J Mol Sci. 2023 Sep 22;24(19):14407. doi: 10.3390/ijms241914407 (PMC10573079; doi:10.3390/ijms241914407)
Supplement: Supplementary file 1 [file ijms-24-14407-s001.zip › Supplemental file S1.pdf]

Overexpressed primer sequence of *L-PRLR* and *S-PRLR*

***L-PRLR*:**GCTAGCGCCACCATGAAGGAAAATGCAGCATCCAGAGTGCTTTTC

ATTCTGCTACTTTTTCTCTTCGCCAGCCTTCTGAATGGACAGTCACCTCCTG

AAAAACCCAAGCTTATTAAATGTCGGTCTCCTGGAAAGGAAACGTTCACCT

GCTGGTGGGAGCCCGGGGCAGATGGAGGACTTCCTACCAATTACACACTG

ACTTACCGCAAGGAAGGAGAAACACTCATCCATGAATGTCCAGACTACAA

AACCGGGGGCCCCAACTCCTGCTACTTTAGCAAGAAGTACACCTCCATATG

GAAGATGTATGTCATCACAGTAAGCGCCATCAACCAGATGGGAATCAGTTC

CTCAGATCCACTTTATGTGGACGTGACTTACATAGTTGAACCAGAGCCTCCT

GTGAACCTGACTTTGGAATTAAAACATCCAGAAGATAGAAAACCATATCTA

TGGATAAAATGGTCTCCACCCACCCTGACTGATGTAAAATCTGGTTGGTTC

AGTATCCAGTACGAAATTCGATTAAAACCTGAGAAAGCAACTGACTGGGA

GACTCATTTTGCTCCAAAGCTGACTCAGCTTAAGATTTTCAACTTATATCCA

GGACAGAAATACCTTGTGCAGATTCGATGCAAGCCAGACCATGGATACTGG

AGTGAGTGGAGCCCAGAGAGCTTCATCCAGATACCTAATGACTTCCCAGTG

AAGGATACAAGCATGTGGATCTTTGTGGGCGTCCTTTCTGCTGTCATCTGTT

TGATTATGGTCTGGGCAGTGGCTTTGAAGGGCTATAGCATGGTGACCTGCAT

CCTCCCACCAGTTCCAGGGCCAAAAATAAAAGGATTTGATATTCATCTGCT

GGAGAAGGGCAAGTCCGAAGAACTTCTGAGAGCTCTGGAAAGCCAAGAC

TTCCTTCCCACTTCTGACTGCGAGGATTTGCTGATGGAATTCATAGAGGTAG

ATGACAGTGAGGACCAACACCTGATGCCACACCCCTCCAAAGAACACATG

GAGCAAGGCGTGAAGCCCATGCACCTGGATCCTGACACTGACTCTGGCCG

GGGCAGCTGTGACAGCCCTTCACTCTTGTCTGAAAAGTGTGATGAACCTCA

GGCCTATCCCTCCAAGTTCCACATTCCAGAGGGCCCTGAGAAGCTGGAGGA

TCCCGAAACAAATCATACATGTCTCCAGGCCCCTCAGAGCACAAGTGGGG

AAGGCAAAATCCCCTATTTTCTGGCCAACGGACCCAAATCTTCCACATGGC

CTTTCCCGCAGCCCCCCAGCCTGTACAGCCCCAGATATTCTTACCACAACAT

TGCTGACGTGTGTGAGCTGGCCCTGGGCATGGCAGGCACCACAGCCACTC

TGCTGGACCAAACAGACCAACATGCCTTTAAACCCTCAAAAACCATTGAG

ACTGGCGGGGAAGGAAAGGCAGCCAAACAGAGCGAGTCAGAAGGCTACA

GTTCCGAGCCTGACCAAGACATGGCATGGCCACTGCTCCAAGACAAAACC

CCCTTGTTCTCTGCTAAACCCTTGGAATATGTGGAGATCCACAAGGTCAGC

CAAGATGGAGTGCTAGCTCTGTTCCCAAAACAAAATGAGAAGGTTGACGC

CCCTGAAACCAGCAAGGAGTACTCGAAGGTGTCTCGGGTGACGGATAGCA

ACATCCTGGTGTTGATACCGGATCTGCAAGCGCAAAACCTGACTCTGTTAG

AAGAATCAGCCAAGAAGGCCCCGCCAGCCCTGCCATAGTCTAGA

S-PRLR:GCTAGCGCCACCATGAAGGAAAATGCAGCATCCAGAGTGCTTTTC

ATTCTGCTACTTTTTCTCTTCGCCAGCCTTCTGAATGGACAGTCACCTCCTG

AAAAACCCAAGCTTATTAAATGTCGGTCTCCTGGAAAGGAAACGTTCACCT

GCTGGTGGGAGCCCGGGGCAGATGGAGGACTTCCTACCAATTACACACTG

ACTTACCGCAAGGAAGGAGAAACACTCATCCATGAATGTCCAGACTACAA

AACCGGGGGCCCCAACTCCTGCTACTTTAGCAAGAAGTACACCTCCATATG

GAAGATGTATGTCATCACAGTAAGCGCCATCAACCAGATGGGAATCAGTTC

CTCAGATCCACTTTATGTGGACGTGACTTACATAGTTGAACCAGAGCCTCCT

GTGAACCTGACTTTGGAATTAAAACATCCAGAAGATAGAAAACCATATCTA

TGGATAAAATGGTCTCCACCCACCCTGACTGATGTAAAATCTGGTTGGTTC

AGTATCCAGTACGAAATTCGATTAAAACCTGAGAAAGCAACTGACTGGGA

GACTCATTTTGCTCCAAAGCTGACTCAGCTTAAGATTTTCAACTTATATCCA

GGACAGAAATACCTTGTGCAGATTCGATGCAAGCCAGACCATGGATACTGG

AGTGAGTGGAGCCCAGAGAGCTTCATCCAGATACCTAATGACTTCCCAGTG

AAGGATACAAGCATGTGGATCTTTGTGGGCGTCCTTTCTGCTGTCATCTGTT

TGATTATGGTCTGGGCAGTGGCTTTGAAGGGCTATAGCATGGTGACCTGCAT

CCTCCCACCAGTTCCAGGGCCAAAAATAAAAGGATTTGATATTCATCTGCT

GGAGATATCACAGCCTTCTCGCCTTGTGTCTGTGTTTTAATCTAGA
