# Supplementary material for: High Prolactin Concentration Induces Ovarian Granulosa Cell Oxidative Stress, Leading to Apoptosis Mediated by L-PRLR and S-PRLR
Source: Int J Mol Sci. 2023 Sep 22;24(19):14407. doi: 10.3390/ijms241914407 (PMC10573079; doi:10.3390/ijms241914407)
Supplement: Supplementary file 1 [file ijms-24-14407-s001.zip › Tables S1-S2.pdf]

Table S1. Primer sequences of mRNAs and sgRNAs

Gene	Sequence 5′-3′	Size (bp)	Tm (℃)	Accession number
Bax	F:TGCTCACTGCCTCACTCACC<br>R:CCCAAGACCACTCCTCCCTA	179	60	XM027978592.1
Bcl-2	F:GATGACCGAGTACCTGAACCG<br>R:GACAGCCAGGAGAAATCAAACA	120	60	XM012103831.3
Caspase3	F:GCTACAAGGTCCGTTATGCC<br>R:GATGCTGCCGTATTCGTTCTC	128	60	XM015104559.2
L-PRLR	F:CCCCTTGTTCTCTGCTAAACCC<br>R: CTATCCGTCACCCGAGACACC	129	60	O46561-1
S-PRLR	F:ACAGTAAGCGCCATCAACCA<br>R:CTGGCTTGCATCGAATCTGC	328	60	O46561-2
GAPDH	F:GGTCGGAGTGAACGGATTTG<br>R:CTTGACTGTGCCGTGGAACTT	222	60	NM001190390.1
L-PRLR-sgRNA	F:Caccg CAAATCCTCGCAGTCAGAAG<br>R:Aaac CTTCTGACTGCGAGGATTTG c			O46561-1
S-PRLR-sgRNA	F:Caccg CTTATTAAATGTCGGTCTCC<br>R:Aaac GGAGACCGACATTTAATAAG c			O46561-2

Table S2. Reactions volume for RT-qPCR

Name	Dose (μl)
Ultra SYBR Mixture (2×)	10
Forward primer (10 μM)	0.4
Forward primer primer (10 μM)	0.4
cDNA template	2
Nuclease-free Water	7.2
Total volume	20
