# Supplementary material for: High Prolactin Concentration Induces Ovarian Granulosa Cell Oxidative Stress, Leading to Apoptosis Mediated by L-PRLR and S-PRLR
Source: Int J Mol Sci. 2023 Sep 22;24(19):14407. doi: 10.3390/ijms241914407 (PMC10573079; doi:10.3390/ijms241914407)
Supplement: Supplementary file 1 [file Figure S1.pdf › Figure S1.pdf]

A B

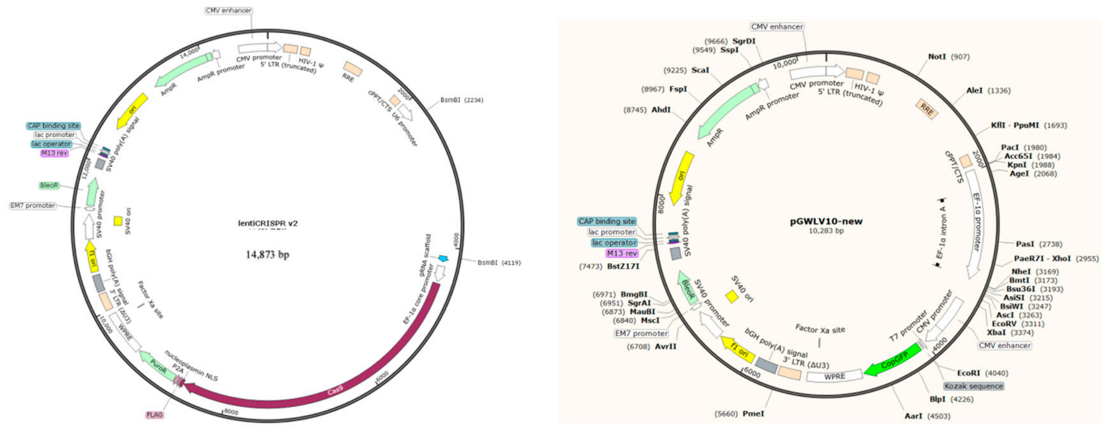

Figure S1 The empty plasmid

A: The empty plasmid lentiCRISPR v2; B: The empty plasmid pGWLV10-new.
